# The complete chloroplast genome of the *Lonicera maackii* (Caprifoliaceae), an ornamental plant

**DOI:** 10.1080/23802359.2019.1710288

**Published:** 2020-01-14

**Authors:** Guolun Jia, Huan Wang, Pei Yu, Peng Li

**Affiliations:** a Key Laboratory of Resource Biology and Biotechnology in Western China, Ministry of Education, School of Life Sciences, Northwest University, Xi’an, People’s Republic of China;; b Xi’an International University, Xi’an, People’s Republic of China

**Keywords:** Chloroplast genome, Caprifoliaceae, *Lonicera maackii*

## Abstract

*Lonicera maackii*, is scattered in west and northeast China as well as adjacent Korea, Japan and the Soviet union. Here, we assembled and characterized the complete chloroplast (cp) genome of *L. maackii* using Illumina sequencing data for the first time. The complete cp genome was 155,337 bp in length, consisting of a pair of inverted repeats of 23,718 bp, a large single-copy region of 89,221 bp and a small single-copy region of 18,680 bp. The genome encoded 113 unique genes, including 79 protein-coding genes, 30 tRNA genes and four rRNA genes. Phylogenetic analysis based on 25 complete cp genome sequences indicated that *L. maackii* is closely related to *Lonicera sachalinensis* and *Lonicera insularis*.

*Lonicera maackii* (Amur honeysuckle) belongs to the Lonicera genus, Caprifoliaceae family. *L. maackii* is a woody perennial shrub which grows up to 5 m in height and sprouts earlier in spring. It is scattered in west and northeast China as well as adjacent Korea, Japan and the Soviet union and has been widely used for ornamental purpose. So far, genetic information of *L. maackii* was barely reported except for karyotype (Chen et al. 2017). The chloroplast genomes have been extensively used in understanding genetic diversity, authentication, and evolution in plants (Joh et al. 2017; Kim et al. 2017; Nguyen et al. 2017; Ye et al. 2014; Nguyen et al. 2018). In this study, we assembled and annotated the complete chloroplast genome sequence of *L. maackii* using for the first time.

Fresh leaves of *L. maackii* were collected from Yinchuan Botanical Garden (38°28′N, 106°16′E; Ningxia, NW China). A voucher specimen (2019LM1) is deposited at the key laboratory of resource biology and biotechnology in Northwest University. We used the modified CTAB method to extract the total genomic DNA (Doyle and Doyle 1987). A shotgun library constructed following the manufacturer’s protocol for the Illumina HiSeq X Ten Sequencing System (Illumina, San Diego, CA, USA). We assembled the cp genome using the program MITObim v1.8 (DSM Nutritional Products Ltd, Kaiseraugst, Switzerland) (Hahn et al. 2013), with that of *Lonicera japonica* (GenBank: *KJ170923*) as the initial reference. To validate the assembly, PCR amplifications and Sanger sequencing were conducted to verify the four junctions between IRs and large single copy region (LSC)/small single-copy region (SSC). The cp genome annotation was performed using DOGMA, coupled with manual correction for protein-coding region (CDS) boundaries. The web-based tool OGDRaw v1.2 (http://ogdraw.mpimp-golm.mpg.de/) was employed to generate a map of the complete cp genome (Lohse et al. 2013).

The complete cp genome sequence of *L. maackii* has been submitted to GenBank (accession number *MN256451*). The complete cp genome was 155,337 bp in length, consisting of a pair of inverted repeat regions of 23,718 bp each, a large single copy region of 89,221 bp, and a small single copy region of 18,680 bp. A total of 113 genes were annotated, including 30 tRNA, 4 rRNA, and 79 protein-coding genes. The overall GC content of the cp genome was 38.5%. In addition, 8 PCG genes (rps16, rps12,atpF, rpoC1, rpl16, ndhA, ndhB and rpl2) possess a single intron, 69 PCG genes no intron, 2 other genes (rps18 and ycf3) harbor two introns. 6 tRNA genes (trnA-UGC, trnI-GAU, trnK-UUU, trnG-UCC, trnL-UAA and trnV-UAC) harbor a single intron.

To reveal the phylogenetic position of *L. maackii* within the Caprifoliaceae, the neighbor-joining method was used by MEGA 7.0 with 1000 bootstrap replicates (Kumar et al. 2016) (http://www.megasoftware.net/). Depending on the available data from GenBank, we selected 25 other complete cp genomes of species from the Caprifoliaceae and three species from Adoxaceae as outgroups. Our result confirmed that the *L. maackii* closely related to *Lonicera sachalinensis* and *Lonicera insularis* (Figure 1).

**Figure 1.**
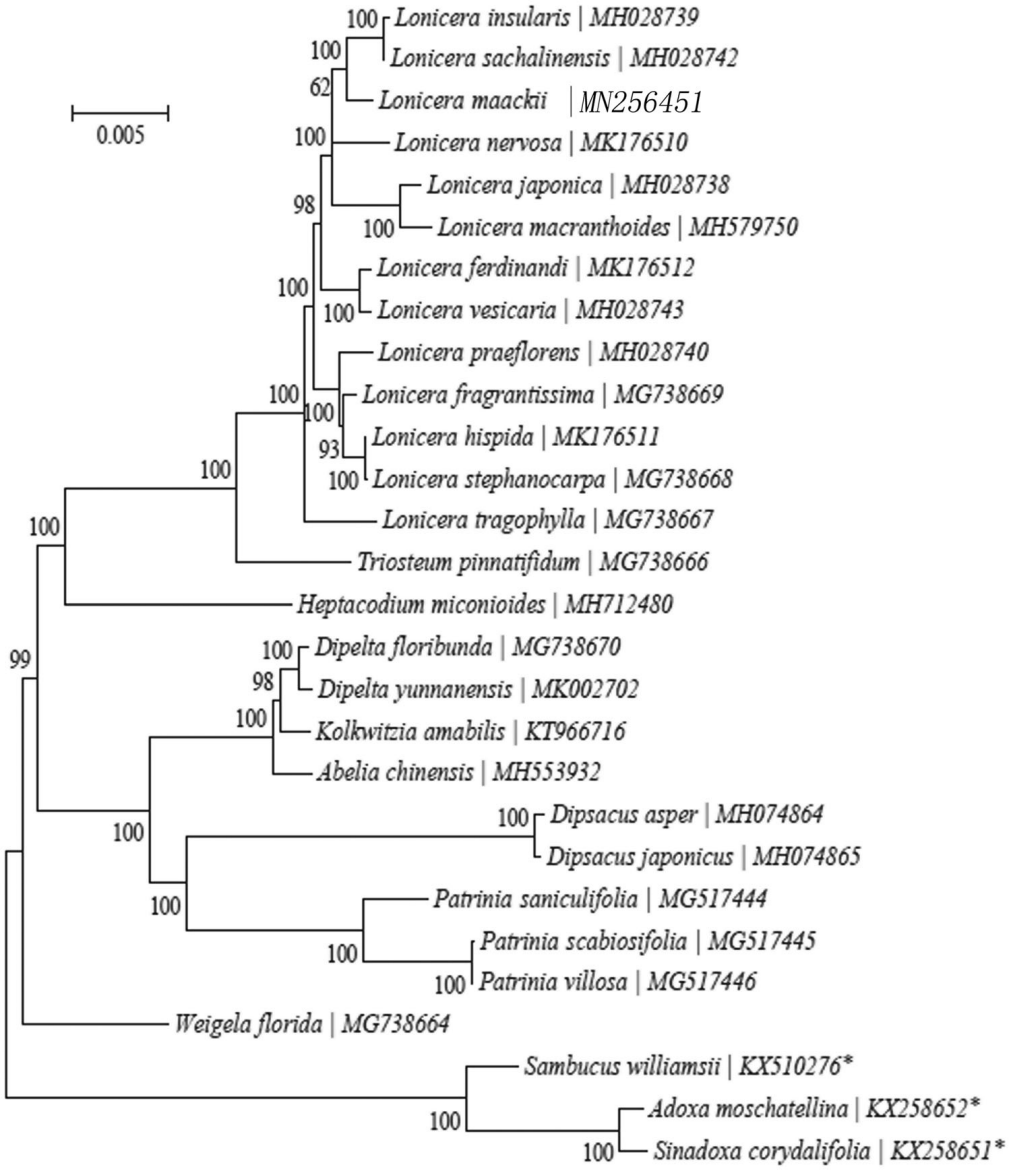
Consensus neighbor-joining tree based on the complete cp sequence of *L. maackii* and other 27 species of Rubiales.
